# Kombucha Tea—A Double Power of Bioactive Compounds from Tea and Symbiotic Culture of Bacteria and Yeasts (SCOBY)

**DOI:** 10.3390/antiox10101541

**Published:** 2021-09-28

**Authors:** Hubert Antolak, Dominik Piechota, Aleksandra Kucharska

**Affiliations:** Faculty of Biotechnology and Food Sciences, Institute of Fermentation Technology and Microbiology, Lodz University of Technology, Wolczanska 171/173, 90-530 Lodz, Poland; 233721@edu.p.lodz.pl (D.P.); 203177@edu.p.lodz.pl (A.K.)

**Keywords:** Kombucha tea, bioactive compounds, functional beverages, phytochemicals, antimicrobial activity, fermentation, acetic acid bacteria, lactic acid bacteria, yeasts, probiotic potential, postbiotic potential

## Abstract

Kombucha is a low alcoholic beverage with high content of bioactive compounds derived from plant material (tea, juices, herb extracts) and metabolic activity of microorganisms (acetic acid bacteria, lactic acid bacteria and yeasts). Currently, it attracts an increasing number of consumers due to its health-promoting properties. This review focuses on aspects significantly affecting the bioactive compound content and biological activities of Kombucha tea. The literature review shows that the drink is characterized by a high content of bioactive compounds, strong antioxidant, and antimicrobial properties. Factors that substantially affect these activities are the tea type and its brewing parameters, the composition of the SCOBY, as well as the fermentation parameters. On the other hand, Kombucha fermentation is characterized by many unknowns, which result, inter alia, from different methods of tea extraction, diverse, often undefined compositions of microorganisms used in the fermentation, as well as the lack of clearly defined effects of microorganisms on bioactive compounds contained in tea, and therefore the health-promoting properties of the final product. The article indicates the shortcomings in the current research in the field of Kombucha, as well as future perspectives on improving the health-promoting activities of this fermented drink.

## 1. From Past to Present

Kombucha tea is a non-alcoholic or low alcoholic, functional beverage obtained in the fermentation of sweetened green or black tea by symbiotic culture of bacteria and yeats (SCOBY). This consortium consists of acetic acid bacteria (AAB), lactic acid bacteria (LAB) and yeasts, and the characteristic feature is cellulose-like pellicle on its surface of the fermentation medium, which results from the metabolic activity of selected stains of AAB [1]. Due to the raw material used for fermentation (tea infusions and plant extracts), as well as the variety of microorganisms, Kombucha is characterized by strong health-promoting properties that attract more and more consumers nowadays.

The first documented references to this drink are dated back to ancient China. The “Divine Che” attributes were appreciated by Tsin Dynasty as early as 220 BC. The beverage has spread to other Far East countries, and in 414 A.D. doctor Kombu brought the tea fungus from Korea to Japan at the behest of Emperor Inkyo to treat his digestive problems. In next centuries, merchants popularized Kombucha in Russia, from where at the turn of 19th and 20th centuries, the “Tea Kvass” disseminated to Europe [2,3]. Consumption of the tea dropped significantly during World War II due to the limited access to the tea and sugar. After the war, starting from Germany, France and Italy the beverage’s popularity increased again [3,4]. Depending on the region of the world to which this drink was brought, the drink gained new names: “Manchurian Mushroom Tea”, “Tea Fungus”, “Kargasok Tea”, “Grib tea kvass”, “Indian Tea Fungus”, “Manchu Fungus”, “Teakwass”, “Tea Beer” and many others. Despite different names, this drink is considered extremely beneficial and pro-health for consumers. Nowadays, Kombucha is one of the most popular fermented beverages with low alcohol content with the fastest growth on the market of functional beverages [4].

All over the world, Kombucha is produced both at homes, handcrafted in small enterprises, and on large, commercial scales. Kombucha Brewers International (KBI) is global non-profit organization of commercial Kombucha companies involved in the global promotion of the drink and protection of producers. Most of the companies involved in the production and distribution of Kombucha are located in North America: 134 companies are based in the United States, 28 in Canada. In Europe, most producers can be found in Spain and the United Kingdom [5]. Overall, there are now over 200 different types of this drink available in the world. Considering the changes resulting from consumer preferences, affecting the plant materials used, as well as AAB, LAB and yeast strains, new types of this drink, characterized by different nutritional, sensory and functional qualities can be expected. Detailed information on Kombucha as a commercial product are described in the review article written by Kim and Adhikari (2020), therefore these topics will be omitted in this review [5]. Even though the global market is dominated by North America, with a share of approximately 52%, the fastest growing region on the world is Asia-Pacific. Globally, the worth of the global Kombucha market in 2019 was USD 1.67 billion, while in 2020 it was approximately USD 2.2 billion, and it is predicted to grow at a revenue-based compound annual growth rate (CAGR) of nearly 20% in years up to 2027 (https://www.expertmarketresearch.com/reports/kombucha-tea-market).

**Figure 1:**
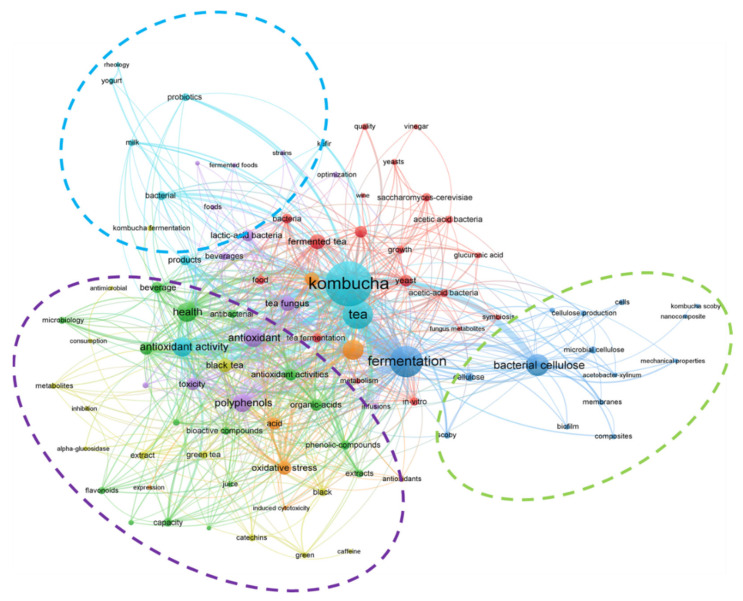
Co-occurrence analysis of the data from Web of Science obtained for “Kombucha” records. Bubble size presents the number of papers in the database. Bubble proximity presents frequency of co-occurrence of phrases in the same papers. The green circle represents bacterial cellulose cluster; blue circle represents probiotic potential and application of SCOBY strains in new products, while purple circle represents health-benefits of Kombucha. Due to numerous interactions, the microbiological cluster (red lines) was not clearly presented.

The analysis of bibliographic data with the use of Web of Science (WoS) and Scopus tools indicates a growing interest in this research area. Web of Science and Scopus were analyzed in June 2021 in order to identify relevant articles, review articles, proceedings papers, book chapters and books published between 2012 and 2021. Simple-searching by “Kombucha” revealed the presence of 349 (WoS) and 535 (Scopus) hits, while combination of “Kombucha” AND (“tea” OR “fermentation” OR “production” OR “analysis” OR “brewing” OR “brewed” OR “fermented” OR “SCOBY”) indicate a total of 326 (WoS) and 493 (Scopus) records, while a combination of “Kombucha” AND (“antioxidants” OR “antioxidant activity” OR “antimicrobial” OR “health” OR “benefits” OR “polyphenols” OR “probiotic” OR “detoxication” OR “cardiovascular” OR “cancer” OR “diabetes” OR “blood pressure” OR “metabolism” OR “cholesterol reduction” OR “anti-inflammatory” OR “antibiotic” OR “anti-aging”) gave 251 (WoS) and 335 (Scopus) records from last 10 years. In general, the number of publications on the production of Kombucha and its biological activities is increasing every year, the number of publications on pro-health activities, the composition of the SCOBY, as well as enhancement of Kombucha with juices and extracts has been growing, in particular. According to the results of the analysis with VOSviewer—a software tool for constructing and visualizing bibliometric networks—several research areas in Kombucha studies can be distinguished: evaluation of fermentation processes, studies on bacterial cellulose, microbiological analysis of Kombucha, as well as application of SCOBY in other food products (Figure 1).

The biological activities and health-promoting properties of Kombucha have been described in several review articles [6,7,8]. The beneficial effects of the beverage results, among others, from antioxidant activities that restore the balance between the production of free radicals and the body’s defense mechanisms. As a result, Kombucha may contribute to the reduction of health disorders such as cancer, cardiovascular diseases, and neurodegenerative diseases. In general, Kombuchas show positive effect on digestion and intestinal microbiota, relief against arthritis, possess antimicrobial activity, relief against hemorrhoids, detoxify body, show hepatoprotective effect, as well as reduce insomnia, relieve headaches, and positively impact a mood [7,8,9]. These activities results from (1) the presence of tea phenolic compounds; (2) phenolic compounds resulting from the metabolic activity of microorganisms (activity of enzymes hydrolyzing tea polyphenols); (3) the presence of organic acids produced by microorganisms; (4) the presence of vitamins from tea leaves or the product of microorganisms; (5) the presence of microbial enzymes and proteins, as well as (6) probiotic activities of microorganisms. On the one hand, teas properties have mainly been documented in in vitro analyses [10,11,12], while in vivo tests are limited among literature [13,14,15]. Most importantly, there are very limited number of clinical studies that would confirm the positive effect of Kombucha [6]. There is one clinical trial on the effect of Kombucha oral administration on glucose metabolism in humans. Parameters such as glucose level and insulin level are being monitored for two control groups (tea infusion, tap water) and two intervention groups (commercial Kombucha and laboratory brewed Kombucha). The study is expected to end in 2021 [6].

The growing popularity of Kombucha, contributes to the use of new materials, strains of microorganisms and fermentation conditions, which results in the changes of the chemical composition, and thus changes of health benefits of its consumption. The dynamic development of this sector of beverages may therefore contribute to the creation of products differing from the concept of functionality. Consequently, it is important to determine the key factors affecting the biological activities of this product, and how the change of the individual components may affect the fermentation process itself, and consequently the health benefits of the final, commercial products.

Despite the fact that there is a relatively large number of review articles on Kombucha, little attention is paid to the identification of bacterial and yeast strains used in SCOBY. Consequently, the influence of specific strains on the health-promoting properties of the product is poorly described. Therefore, in this review, we raise the important issue of mutual interactions between SCOBY microorganisms, parameters of the technological process and product quality.Additionally, based on the literature review, we indicate what further research should be conducted to improve Kombucha.

## 2. Production of Kombucha Tea

### 2.1. Tea Preparation and Influence on the Bioactive Compounds

**Figure 2:**
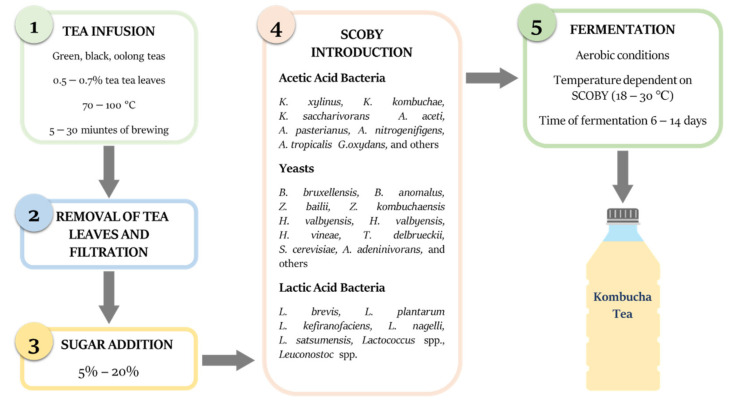
Main stages of Kombucha tea production along with data ranges used in various scientific research or fermentation industry.

Kombucha is traditionally produced from green, oolong or more commonly black tea (Figure 2). The production of Kombucha starts with tea preparation of infusion. To the 1 L of boiling tap water, usually 5 g of tea leaves are added and allowed to infuse with initial temperature between 70 and 95 °C [16]. After removing the leaves by filtration, from 50 to 150 g/L of sucrose is dissolved in the hot tea. Before introducing the tea fungus, the infusion is cooled down to about 20 °C.

In general, black tea (post-fermented), oolong tea (semi-fermented) and green tea (non-fermented) are the main teas used in Kombucha fermentation. Both the composition of bioactive compounds and the resulting biological activities are dependent on the type of tea, but so far, it has not been clearly established how the type of tea influences the time of fermentation by specific strains of SCOBY. Among the above-mentioned, green tea is characterized by a higher content of polyphenols compared to black tea and oolong, and therefore stronger health-promoting properties resulting from the content of epigallocatechin-3-gallate (EGCG)—one of the strongest antioxidants in Kombucha [17]. Zhao et al. (2019) demonstrated that green tea contains more phenolic compounds than yellow, oolong, dark, black and white teas. Green tea was also characterized by the strongest antioxidant properties among the tested teas [18]. Jakubczyk et al. (2020) found that red and green teas are richer source of polyphenols than back tea, thus may be a more attractive alternative than Kombucha obtained with black tea [19].

In addition to the type of tea used for Kombucha fermentation, the parameters of tea brewing, time and temperature, have a significant impact on the content of bioactive compounds and antioxidant activities [20,21]. It is known that temperature and time of the brewing are the main factors affecting extraction of catechins from tea, especially when cold extraction is used [22]. According to the results of Pérez-Burillo et al. (2018) infusion time plays crucial role in the extraction of bioactive compounds, but temperature from 60 to 80 °C do not play any significant role in brewing time shorter than 15 min. Higher concentration of gallic acid, epigallocatechin and caffein were noted in infusions obtained at temperature higher than 80 °C. Authors declared that temperature of 98 °C with brewing time 7–15 min increases the content of bioactive compounds and thus antiradical and reducing capacity [23]. These data are in line with results obtained by Zimmermann and Gleichenhagena (2011), authors found that the concentrations of flavanols in green tea are the highest after 7 min of steeping at a constant 100 °C [24]. Saklar et al. (2015) observed rapid increase of catechins for the first 5 min at 85 °C [25]. Phenolic profiles and antioxidant activities of oolong tea extract were investigated by Su et al. (2007) and the strongest antioxidant activity were ascribed to 100 °C for 3 min, while longer time of extraction led to decrease of the polyphenols [26]. Relatively extensive research on the influence of factors on the properties of tea was carried out by Sharpe et al. (2016). Authors concluded that infusions with greater antioxidant capacity are obtained at temperatures from range of 80 to 100 °C extracted for 5 to 10 min [27].

In addition to the brewing time and temperature, the quality of the water used for the extraction of bioactive compounds from tea is also important for the composition of the infusions obtained. In studies conducted by Xu et al. (2017) the concentrations of catechins were significantly influenced by the water used for brewing. Application of mountain spring water and purified water resulted in higher catechin concentrations than in the case of mineral water and tap water. It is known that stability of phenolic compounds is pH-dependent, and catechin is unstable in aqueous solutions with pH > 6 [28]. What is more, the extracts obtained with purified water were characterized by higher concentrations of caffeine and amino acids [29].

To obtain Kombucha tea with more attractive sensory values or improved health-promoting properties, as well as to develop new products, innovative alternative materials are used. One of the most recent reviews written by Emiljanowicz and Malinowska-Pańczyk (2020) broadly describes Kombucha-based functional beverages from leaves, herb infusions, vegetable pulp, fruit juices, and others [30]. In general, the addition of apple juice, guava (*Psidium guajava* L.), *Malvaviscus arboreus* Cav., kitchen mint (*Mentha cordifolia* Opiz. Ex Fresen) [31], cinnamon [32], date syrup [33] can increase antioxidant activity of the final product. *Brassica tournefortii* Gouan. fermentation using SCOBY was found to increase total phenolic content, increase antioxidant activity, reduce cytotoxicity [34]. Application of pollen can induce the production of postbiotics such as short chain fatty acids (SCFA), which are characterized by inter alia immunomodulatory, hypocholesterolemic, and antioxidant activities [35]. Sensory characteristics and antioxidant activities of sweetened oak herbal infusions (*Quercus resinosa* Liebm.) fermented with Kombucha consortium were noted in the studies of Vázquez-Cabral et al. (2014) and Vázquez-Cabral et al. (2017) [36,37]. Anthocyanin-rich Zijuan tea was proposed as an attractive alternative to Kombucha from green or black tea [38]. It is believed that new products containing innovative additives aimed at pro-health properties, increasing sensory values, or simply encouraging consumers, will appear on the market. However, it should be remembered that with the use of new materials for Kombucha fermentation, new chemical compounds are introduced, the activity of which in relation to SCOBY is not fully known. Moreover, the research data on the selection of strains of microorganisms present in SCOBY and their adaptation to the new environment (enriched with other materials than teas) are limited and constitute the prospect of further research.

### 2.2. SCOBY Composition and Metabolic Acticity

Kombucha tea is considered as a reservoir of specialized microorganisms that are rapidly changing in terms of the quantitative and qualitative composition [39]. Depending on the region of the world, used raw materials, the conditions of the fermentation, different genera, species and strains of acetic acid bacteria, lactic acid bacteria and yeasts are identified in SCOBY [8]. Taking into account the metabolic activity of various strains of microorganisms, the obtained products may be characterized by various composition: the content of organic acids, vitamins, enzymes, and antioxidant activity [40,41].

**Figure 3:**
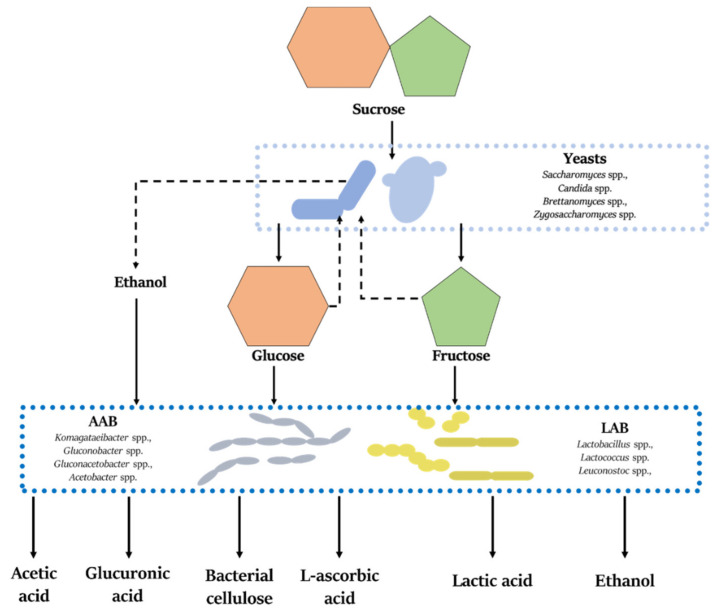
Scheme of sucrose metabolism by SCOBY along with major metabolites.

Within the SCOBY consortium, a number of symbiotic interactions occurs, and due to their complex nature and interactions between microorganisms and the fermentation environment, they are a subject of a number of research studies. One of the most important and well-documented, is the utilization of sucrose. As a result of invertase (β-fructofuranosidase, EC 3.2.1.26) activity, yeast strains hydrolyze sucrose, releasing glucose and fructose, which are substrates for the production of further metabolites (Figure 3). In general, *Saccharomyces* spp. use glucose and produce ethanol via glycolysis, while *Zygosaccharomyces* spp. produce EtOH more efficiently from fructose. What is more, specific strains of yeasts can produce ethanol from malic acid (*Schizosaccharomyces pombe*), or high concentration of acetic acid in aerobic conditions (*Brettanomyces bruxellensis*) [42]. Among the microorganisms belonging to the SCOBY, yeasts are considered to be the main producers of ethanol. In turn, the obtained ethanol is oxidized by AAB to acetic acid. At the same time, *Komagataeibacter* spp. use glucose to synthesize bacterial cellulose, but ethanol, sucrose or glycerol also take part in the synthesis [12,43]. In addition to acetic acid and bacterial cellulose, AAB are responsible for glucuronic acid (GlucUA) production. This uronic acid is produced from glucose, which is metabolized to gluconic acid and then converted to glucuronic acid which bring consumers numerous health benefits [12,44]. Additionally, *Gluconobacter* strains can synthetize vitamin C (L-ascorbic acid) from D-sorbitol, which in turn obtained from glucose [45]. Depending on the specie, LAB can use glucose in the Embden–Meyerhof–Parnas pathway, where lactic acid is obtained as the main metabolite (homofermentative LAB), or through the pentose phosphate pathway, which results in the synthesis of lactic acid, ethanol, and carbon dioxide (heterofermentative LAB). In the case of fructose, acetic acid is produced instead of ethanol [46]. Other compounds of bacterial or yeast found in Kombucha tea are described in later parts of this article.

In general, species belonging to *Komagataeibacter* (*K. xylinus*, *K. kombuchae*), *Acetobacter* (*A. aceti*, *A. pasteurianus*, *A. nitrogenifigens*), *Gluconacetobacter* (*G. sacchari*) are major AAB strains, while strains of *Saccharomyces* (*S. cerevisiae*) and non-*Saccharomyces* (*Candida* spp., *Schizosaccharomyces* spp., *Dekkera* spp., *Brettanomyces* spp.) are leading yeasts representatives [30,47,48]. Despite the large number of studies using SCOBY, a relatively large number of articles do not provide clear data on the bacterial and yeasts composition of the used consortium [34,49,50,51,52]. Although it is necessary to conduct further research on the particular groups of microorganisms, and even specific species, as well as their role in Kombucha fermentation.

Coton et al. (2017) found that *Komagataeibacter europaeus*, *Gluconobacter oxydans*, *Komagataeibacter saccharivorans* and *Acetobacter peroxydans* are dominant acetic acid bacteria, *Brettanomyces bruxellensis*, *Brettanomyces anomalus*, *Zygosaccharomyces bailii* and *Hanseniaspora valbyensis* are predominant yeasts strains during Kombucha fermentation, while *Oenococcus oeni* was associated with green tea fermentation [53]. Sequence based analysis of commercial starter of Kombucha showed that *Pichia* and *Dekkera* (*Brettanomyces*) were the dominant yeast species, while *Acetobacter* and *Lactobacillus* were the key bacterial genera in the commercial starter of Kombucha investigated by Tu et al. (2019). In this study, the consortium was used in order to obtain soy whey-based fermented beverage [54]. In other studies, SCOBY strains were used to create a novel functional dairy product but bacterial and yeast names were not specified [50]. Rahmani et al. (2019) used SCOBY in the fermentation of *Brassica tournefortii* Gouan leaves, but also in the case of this study, the composition of the consortium was not described [34]. What is more, *Hanseniaspora valbyensis*, *Hanseniaspora vineae*, *Torulaspora delbrueckii*, *Zygosaccharomyces bailii* and *Zygosaccharomyces kombuchaensis* isolated form Kombucha were applied in the production of an alcohol-free beer [55]. On the other hand, yeast ecology was also investigated by Teoh et al. (2004). The diversity of species varied between tested samples, but generally included *Z. bailii*, *T. delbrueckii*, but also *Brettanomyces bruxellensis*, *Candida stellata*, *Schizosaccharomyces pombe* were identified [56]. *Dekkera bruxellensis* (synonym: *Brettanomyces bruxellensis*) was described in the study of Nguyen et al. (2015) [57].

Studies on five Kombucha starters were conducted by Marsh and co-workers (2014). Sequence-based analysis showed that *Gluconacetobacter* spp. was the major bacterial genus present in most samples (more than > 85% share), while *Acetobacter* was detected at level less than 2%. It should be highlighted that *Gluconacetobacter* is a formal name of some species classified to *Komagataeibacter* genus. *Zygosaccharomyces* spp. (*Z. lentus* and *Z. bisporus*) were major species of yeasts in the tested samples of Kombucha. Additionally, *Dekkera* and *Kazachstania* were also detected in fermented tea samples. *Lactobacillus kefiranofaciens*, *Liquorilactobacillus nagelii* (formerly *Lactobacillus nagelii*), *Liquorilactobacillus satsumensis* (formerly *Lactobacillus satsumensis*), *Lactococcus* and *Leuconostoc* species were also identified in the study [58]. It is known that lactic acid bacteria and their products show high antioxidant activity [59,60,61]. A strain of lactic acid bacteria has been used successfully to ferment green tea leaves in research conducted by Jin et al. (2021). Application of selected *Levilactobacillus brevis* (formerly *Lactobacillus brevis*) strain caused significant increase in DPPH activity from 88.96% to 94.38% after the fifth day of fermentation. It is worth noting that the strain was characterized by high production of γ-aminobutyric acid (GABA) and authors concluded that initial DPPH of tea result from the content of polyphenols, while GABA and other bioactive compounds may contribute to the activity of the fermented product [62]. Although some studies indicates that LAB are present in low numbers in the Kombucha. In the first days of fermentation an increase in their number is observed, but then a decrease and then a gradual decline in population occurs [63]. Therefore, it is possible that inoculation of tea infusion with SCOBY from a previous fermentation lasting more than 14 days may result in absence of lactic acid bacteria during a new process.

Shotgun sequencing conducted in the study of Villarreal-Soto et al. (2020) revealed that almost 80% of the microorganisms from Kombucha samples belonged to *Acetobacteraceae*, (*Komagataeibacter* spp., *Gluconacetobacter* spp., and *Gluconobacter* spp., *Acetobacter malorum*, *A. pasteurianus*, *A. pomorum*, and *A. tropicalis*) [15]. Among acetic acid bacteria *Komagataeibacter rhaeticus* was the dominant bacteria in the matrix, while *Komagataeibacter xylinus* in the liquid phase. In the same study authors found *Candida arabinofermentans*, *Brettanomyces bruxellensis*, *Schizosaccharomyces pombe* and *Zygosaccharomyces bailii*. On the other hand, Zhao and Sui (2018) identified *Saccharomyces cerevisiae* and *Arxula adeninivorans* as major yeast strains, while *Komagataeibacter saccharivorans* (formerly *Gluconacetobacter saccharivorans*), *Acetobacter* sp., *Komagataeibacter europaeus* (formerly *Gluconacetobacter europaeus*), *Acetobacter aceti* and *Limosilactobacillus fermentum* (formerly *Lactobacillus fermentum*) where found as major bacterial representatives of Kombucha starter [64]. In the latest research on over 100 samples of Kombucha tea from North America, *Komagataeibacter* (*K. europaeus*, *K. medellinensis*, *K. saccharivorans*, *K. nataicola*, *K. xylinus*, *K. rhaeticus*), *Acetobacter* (*A. senegalensis*, *A. tropicalis*), *Gluconobacter* (*G. oxydans*), and *Liquorilactobacillus nagelii* were described as main bacteria, while *Brettanomyces* (*B. bruxellensis* and *B. anomalus*), *Saccharomyces cerevisiae*, *Saccharomycodes ludwigii*, *Zygosaccharomyces bisporus*, *Starmerella davenpoortii*, *Schizosaccharomyces pombe* were identified as yeast strains [65].

Studies conducted by Gaggìa et al. (2019) showed similarities in bacterial composition among green, black and rooibos tea fermentation, with the dominance of *Komagataeibacter* spp. (*Komagataeibacter rhaeticus*). *Gluconacetobacter entanii* was detected almost exclusively in Kombucha from rooibos. On the base of high-throughput sequencing of 16S rRNA gene *Acetobacteraceae* was found as a major family of bacteria, with *Komagataeibacter* spp. as a dominant genus, but *Lactobacillaceae*, *Paenibacillaceae*, *Staphylococcaceae*, *Streptococcaceae*, *Lachnospiraceae* were also detected. Among yeasts, *Pichiaceae* (*Brettanomyces bruxellensis*) and *Saccharomycetaceae* (*Zygosaccharomyces parabailii*) representatives were found on the base of sequencing of ITS region [1].

An important aspect from the point of view of health-promoting properties of Kombucha tea is its potential and probiotic nature, which results from the presence of living microorganisms [66]. In addition to lactic acid bacteria and yeast, recent studies state that acetic acid bacteria can be an alternative to lactic acid bacteria in this area [67]. According to the definition, probiotics are “live microorganisms which when administered in adequate amounts, confer a health benefit on the host” [68] and can be successfully applied in fermented food products [69]. On the other hand, after prolonged fermentation or storage, as well as pasteurization of the products, fermented products might contain substantial number of non-viable cells. What is more, non-viable microorganisms, as well as their components and end-products play a part in the health benefits of fermented products, including plant-based such Kombucha. According to the latest statement of The International Scientific Association of Probiotics and Prebiotics (ISAPP) “preparation of inanimate microorganisms and/or their components that confers a health benefit on the host” is the definition of postbiotics [70]. Taking into account the fact that Kombucha fermentation involves three groups of microorganisms with different quantitative and qualitative compositions, which release various metabolites (vitamins, organic acids); as well as the fact that a significant number of cells are lysed, as a result of either complex succession of species, or changes in physicochemical conditions, Kombucha might be a reservoir of probiotic strains or considered as postbiotic beverage. However, to be able to name this drink as “postbiotic” accurate identification of the strains used in the fermentation, description of the inactivation procedure, as well as quantification of the final postbiotic composition, are needed [70]. As noted above, Kombucha may be a source of potentially probiotic microorganisms, but research on the postbiotic potential has not yet been carried out and constitutes a specific further perspective in this area.

### 2.3. Fermentation

After the preparation of sweetened tea and SCOBY starter, the infusion is cooled down to about 20 ℃, and 1–10% of previously fermented Kombucha is added to the fermentation medium. In order to ensure oxygen conditions and at the same time to protect against insects or contamination microbiota, bioreactor is covered with fabric (traditional fermentation), unsealed covers of reactors or additional aeration are also applied. The optimal temperature for Kombucha fermentation ranges from around 20 to 30 °C, but the temperature dependent on the SCOBY composition [3]. A few days after the start of fermentation, the formation of new tea fungus floating on the surface of beverage, in the form of thin jelly membrane is observed. The fermentation is carried out from 7 to 60 days, or until the pH drops to about 4.2 [8]. On the other hand, Food and Drug Administration (FDA) recommends a fermentation up to 10 days [53]. Watawan et al. (2015) indicated that long-term fermentation may cause excessive acidification of the beverage due to the metabolism of AAB and LAB, which could reduce the health-promoting properties of the drink or even adversely affect the body of consumers [7]. When the fermentation is completed, the SCOBY is removed and subsequently, Kombucha is filtered.

Parameters such as the type of tea, sugar content, fermentation temperature and its fermentation time can significantly affect the characteristics of the obtained product, including health-promoting. Ivanišová et al. (2020) reported that Kombucha after seven-days fermentation at 22 °C (no information on the SCOBY composition), was characterized by higher polyphenols (412.25 mg GAE/L) than black tea infusion (180.17 mg GAE/L). What is more, the antioxidant activities of the obtained beverage were also higher: 1318.56 mg TEAC/L (Kombucha) and 345.59 mg TEAC/L (tea) [71]. Jakubczyk et al. (2020) evaluated antioxidant activity and total phenolic content (TPC) of Kombucha obtained by the fermentation (28 ± 1 °C for up to 14 days, no specific composition of SCOBY) of green, black, white and red teas. Authors found that antioxidant activities and TPCs of Kombucha depends on the type of tea as well as time of fermentation. For example for green tea after 7 days of fermentation TPC equaled 299.6 ± 3.1 mgGAE/L, while after 14 day 320.1 ± 3.5 mgGAE/mL was noted. On the other hand, for black tea TPC values equaled 219.5 ± 2.1 and 206.0 ± 1.2 mgGAE/L were noted for 7 and 14 days, respectively. In addition, antioxidant activities depended on the time fermentation [19]. Diversified values of TPC and antioxidant properties were described in the work of Gaggìa et al. (2019) [1]. The authors noted lower polyphenol content in Kombucha (27 ± 1 °C for 14 days) compared to tea infusions. TPC for green tea infusion was 74.40 ± 1.64 mg/g DW, after 7 days of fermentation it increased up to 100.33 ± 2.36 mg/g DW, while after 14 days of fermentation, a decrease to 67.40 ± 2.69 was noted. As in other articles, the values of antioxidant activity varied between the tea and fermentation time. In general, it is believed that catechins and other phenolic compounds are responsible for the antioxidant properties of Kombucha as they could easily give a hydroxyl hydrogen, and the antioxidant activities are stronger in products after shorter fermentation [72]. Chu and Chen (2006) noted increasing concentrations of phenolic compounds and antioxidant activities (DPPH, ABTS) during the fermentation (30 °C for 15 days) of various samples of black tea [73]. On the other hand, Ahmed et al. found that phenolic compounds and antioxidant activities of Kombucha from black tea (28 ± 2 °C, 12 days) increase to the eighth day of fermentation, and then the values slowly decrease [11]. Similar observation on the initial growth and further decrease of TPC and antioxidant activities were noted by Ayed et al. (2017) [63].

It is known that the microorganisms involved in Kombucha fermentation may exhibit metabolic activity in a wide range of temperatures, but the most common fermentation temperature are 20–30 °C. In general, optimum growth temperature for acetic acid bacteria is between 25 and 30 °C [45], while for *K. xylinus* the values ranged from 18 to 22 °C [74]. Some strains of AAB are able to show metabolic activity at temperatures close to 10 °C. The best growth of *Brettanomyces bruxellensis* is observed between 22–32 °C, while for *Zygosaccharomyces rouxii* and *Saccharomyces cerevisiae* optimal temperatures are 30 °C and 32 °C, respectively [75]. What is more, lactic acid bacteria growth temperature ranges between 20 and 40 °C, but *Lactobacillus* strains are able to grow at lower temperature such as 8 °C [76]. It is believed that both the antioxidant properties and the polyphenol content depends on the composition of the microorganisms in SCOBY. Malbaša and others (2011) used different consortia for black and green tea fermentation (28 °C, 10 days). (1) AAB and *Zygosaccharomyces* sp.; (2) AAB and *Saccharomyces cerevisiae*; as well as (3) native local Kombucha. For green tea fermentation, the “native SCOBY” was the most prominent, although DPPH values decreased with the duration of fermentation. In the case of black tea, AAB in the consortium with *Zygosaccharomyces* sp. contributed to obtaining the product with the strongest antioxidant properties, although the activity also decreased with the fermentation time [77]. On the other hand, the fermentation temperature essentially influences the metabolic activity of microorganisms, resulting in products with different sensory characteristics as well as production of bioactive compounds [78]. Neffe-Skocińska et al. (2017) found that optimal temperature of Kombucha fermentation was 25 °C. The content of glucuronic acid in product obtained at 25 °C was higher than at 20 °C or 30 °C [79]. According to Thavasi et al. (2009) temperature is the major factor that affects the antioxidant activity during fermentation [80]. This factor has a major influence on the activity of the enzyme (β-glucosidase) involved in the hydrolysis of phenolic glycosides and the release of free aglycones, which are characterized by high antioxidant activity [81]. Higher temperature results in greater kinetic energy, and as a result OH group of the phenols becomes more labile, and the H atom readily dissociates [80]. Increased concentration of polyphenols were noted for bush tea (*Athrixia phylicoides* DC.) fermented at 30 °C, followed by fermentation temperatures of 34 °C and 38 °C, while temperature of 24 °C and 42 °C decreased the bioactive compounds [82]. The effect of temperature on the bacterial community and TPC during Kombucha fermentation was evaluated by Filippis et al. (2018) [83]. Higher temperature of fermentation (30 °C) resulted in greater diversity of bacteria than fermentation at 20 °C. Authors noted higher concentration of gluconic and glucuronic acids at higher temperature, and this was correlated with the levels of *Komagataeibacter saccharivorans*. What is more, it was found that TPC values increased in black tea during the fermentation at 20 °C, while at 30 °C total polyphenols significantly decreased after 15 days [83]. However, information regarding the influence of temperature on the health-promoting properties of Kombucha is extremely limited and more in-depth research are needed.

## 3. Bioactive Compounds

**Figure 4:**
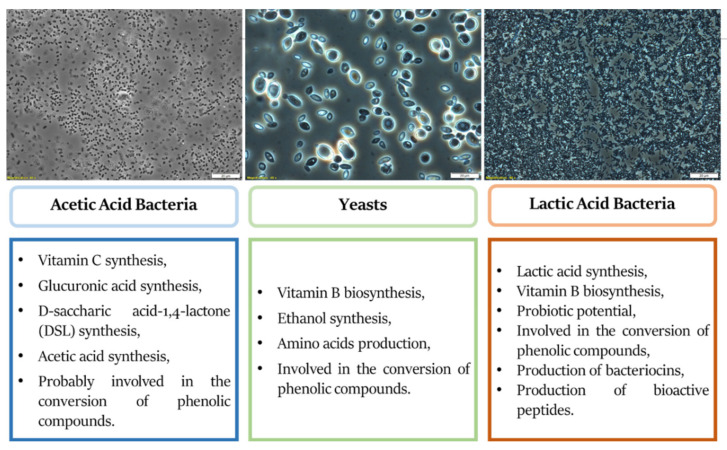
The influence of the SCOBY consortium on the health-promoting properties of fermented tea—Kombucha.

The bioactive compounds contained in Kombucha can come both from tea (phenolic compounds, polysaccharides, vitamins, minerals, amino acids), as well as from the metabolic activity of microorganisms involved in the fermentation of this beverage. Bioactive compounds obtained with the participation of microorganisms include polyphenols (resulting from the metabolic activity of SCOBY), organic acids, vitamins, enzymes, proteins such as bacteriocins (Figure 4). The final health benefits of the product depend on the plant material, as well as the consortium of microorganisms used for fermentation.

### 3.1. Phenolic Compounds

Main phenolic compounds of green tea includes flavanols, flavonols and phenolic acids. Flavanols (gallocatechin (GC), catechin gallate (CG), gallocatechin gallate (GCG), epicatechin (EC), epigallocatechin (EGC), epicatechin gallate (ECG) and epigallocatechin gallate (EGCG)) are the most abundant among green tea phenolic compounds, accounting for more than 70% of TPC [84].

Flavonols in green tea include glycoside structures (glucose, galactose, rhamnose, rutin) of kaempferol and quercetin [85], while among phenolic acids gallic, *p*-coumaric, quinic acid derivatives, caffeoylquinic acid isomers are reported [84]. Zhao et al. (2019) identified six main catechins in green tea: EGCG, EGC, ECG, EC, GC, GCG, and the highest content was found for EGCG (48.48 mg/g DW) [18]. What is more, epicatechin, epicatechin gallate and epigallocatechin gallate were detected in selected black teas, while in oolong teas EC, GC, EGC, ECG, GCG and EGCG were detected. Among the phenolic acids, gallic and ellagic were detected in the majority of the samples. The concentration of gallic acid ranged from 0.29 ± 0.02 mg/g DW (oolong tea) to 3.77 ± 0.32 mg/g DW (black tea), while ellagic acid concentration ranged from 2.10 ± 0.20 mg/g DW (oolong) to 7.77 ± 0.70 mg/g DW (green tea). The highest contents of caffeine were detected in green tea, yellow tea, and black tea [18].

In general, the concentration of total catechins is higher in green tea than in oolong, white tea or black tea [86]. Although, data obtained by Yi et al. (2015) show that yellow tea (118.55 mg/g) is richer source of EGCG than green tea (112.72 mg/g) [87]. What is more, authors found that the concentration of EGC, EC, ECG, GCG, GC are higher in green and yellow teas than in black and oolong. What is more, quercetin and myricetin were detected in higher concentration in green tea than in black tea while black tea was found to be a better source of kaempferol [88].

Although, secondary metabolites of fresh tea leaves consists mostly of flavan-3-ols, phenolic acids, purine alkaloids, condensed and hydrolysable tannins, as well as saponins, flavonols, and their glycosides [89], the processes (fermentation, postfermentation, roasting) to which tea leaves are subjected, result in the formation of new groups of compounds: theaflavins and thearubigins. The concentration of theaflavin range from 0.66–3.63 mg/g (oolong) to 10.70–17.28 mg/g (black tea) [90]. In the study of Zhao et al. (2019) theaflavin was detected only in black tea samples in the concentration ranging from 0.66 ± 0.06 to 0.70 ± 0.08 mg/g DW [18]. Thearubigins, that are deep oxidation products of catechins and theaflavins are major components of the color of black tea infusions and consists about 20% of total solids of black tea [89,91]. In general, theaflavins can undergo further oxidation during the fermentation of black or Pu-erh teas and form thearubigins, and further condensed theabrownins [92,93]. It is believed that theabrownin is the main bioactive compound in Pu-erh tea with the content of 100–200 g/kg, while in black tea the concentration of the compounds ranges from 100 to 140 g/kg [94].

The composition of bioactive compounds in fermented products affects their antioxidant activity and further health-benefits. The initial content of bioactive compounds that derives from tea may be changed due to the metabolic activity of microorganisms during fermentation. Black tea Kombucha’s polyphenols after 21 days of fermentation at 28 °C was studied by Bhattacharya et al. (2016) [95]. Authors found that the contents of total polyphenols and flavonoids increases in Kombucha compared to the sweetened black tea [95]. This can be a result of degradation of the complex compounds into smaller ones. Consequently, the content of phenolic compounds in Kombucha is increased. In the study of Jafari et al. (2020), black tea was fermented for 14 days in room temperature (*Komagataeibacter* spp., *Lactobacillus* spp., *Saccharomyces cerevisiae*, *Hanseniaspora uvarum*, *Dekkera* spp.). Authors found that increasing activity of invertase during fermentation, results in the increased content of phenolic compounds and thus stronger antioxidant activities [96]. According to Cardoso et al. (2020) Kombucha obtained (25 °C for 10 days, no specific information on SCOBY composition) from black tea shows stronger antioxidant activity prior to Kombucha from green tea [97]. Authors identified 103 phenolic compounds reported for the first time in fermented tea. According to their results, the change in phenolic compounds during the fermentation is more evident for black tea Kombucha, and the obtained product shows greater diversity of phenolic compounds, which can be a result of higher concentration of polymeric phenolic compounds in black tea. Changes in the composition of phenolic compounds during the fermentation of Pu-erh tea (28 °C, 14 days) were also studied by Zhao, et al. (2018). In their study SCOBY composed of *K. saccharivorans*, *Acetobacter* sp., *Gluconacetobacter* sp., *K. europaeus*, *A. aceti* and L. fermentum, *S. cerevisiae* and *Arxula adeninivorans*, was used. After the fermentation, authors noted a decrease in the concentration of total catechins (from 0.572 ± 0.008 mg/mL to 0.322 ± 0.016 mg/mL), and simultaneous increase in the concentration of EGC (from 0.031 ± 0.005 to 0.041 ± 0.001 mg/mL) and EC (from 0.011 ± 0.002 mg/mL to 0.027 ± 0.006 mg/mL). What is more, the concentration of caffein was also decreased after 14 days of fermentation [64].

It is know that some strains, in particular lactic acid bacteria, are able to degrade phenolic compounds. Jayabalan et al. (2007) noted epicatechin degradation to vanillic acid during black tea fermentation [98]. In the study of Vázquez-Cabral et al. (2014) lower contents of catechin, epicatechin, hydroxybenzoic acid derivatives (ellagic) hydroxycinnamic (chlorogenic acid) and higher concentrations of vanillic, benzoic, protocatechuic, gallic acid as well as caffeic acids were noted for functional beverage obtained from infusions of oak leaves [36]. After the fermentation of guava, the content of catechin, gallocatechin was lower than in traditional Kombucha tea, but the concentration of these compounds increased with the time of fermentation. What is more, higher concentration of dicaffeoyl quinnic acid derivates at the beginning of the fermentation was noted for traditional Kombucha, while the presence of dicaffeoyl quinic acid at the beginning of the fermentation of guava was lower, then increased during the process, and finally decreased. Authors concluded that research on the metabolic changes of hydroxycinnamic acids during Kombucha fermentation and SCOBY influence, in particular *Saccharomyces cerevisiae*, are needed [99]. What is more, ferulic acid, *p*-coumaric acid, and caffeic acid can be transformed by *Brettanomyces anomalus* by hydroxycinnamic acid decarboxylase to hydroxystyrenes [100]. It is also worth noting that, in addition to the determination of the effect of individual strains on the phenolic components of Kombucha, it is necessary to determine the effect of phenolic compounds on microbial activity—especially when new SCOBY consortia are designed.

### 3.2. Carbohydrates and Amino Acids

In general, tea polysaccharides contain 44% of neutral sugar and 43% of uronic acid.. Neutral polysaccharides, contains 83% total sugar, 12.9% of which is uronic acid, while acid polysaccharides contains approximately 86% total sugar, 39.8% of which is uronic acid. Rhamnose, arabinose, galactose, glucose, xylose, mannose, ribose, galacturonic acid, and glucuronic acid are main basic unit of tea polysaccharides. Galactose is main component of neutral polysaccharides, while rhamnose, arabinose, galactose and galacturonic acid are found in acid polysaccharides [101]. Polysaccharides’ profiles are influenced by tea cultivars, growth environment as well as processing. Galacturonic acid (65%), arabinose (19%), galactose (7%), glucose (7%) and rhamnose (2%) are found for green tea, while galacturonic acid (35%), arabinose (30%), galactose (16%), rhamnose (3%) and glucose (16%) for black tea. Tea polysaccharides composed of arabinose, ribose, galactose reduce bloody fat, while those composed primarily of galactose show hypoglycemic activity [102].

Although the saccharides contained in tea leaves can affect both the fermentation process of Kombucha and its health-promoting properties, data on saccharides’ profiles in fermented tea are limited to the content of sucrose, fructose and glucose—the main sugars used by microorganisms in SCOBY. Depending on the invertase activity, and thus the composition of the consortium in terms of yeast strains, glucose and fructose release may be different. This affects further metabolites, such as the content of organic acids, as well ethyl alcohol. For example, glucose is metabolized by homofermentative LAB via Embden–Meyerhof–Parnas (EMP) pathway to lactic acid, while heterofermentative LAB metabolize glucose via pentose phosphate pathway, and lactic acid, EtOH as well as CO_2_ are the main metabolites. On the other hand, in the presence of fructose, instead of ethyl alcohol, acetic acid is produced, and fructose is reduced to mannitol [103]. In general, fructose is preferred as carbon source by yeast cells, while AAB prefer glucose [104]. Overall, the type of sugar used in Kombucha fermentation influences the metabolism of the microorganism consortium, the interactions between the microorganisms and, consequently, the profile of the metabolites obtained.

### 3.3. Organic Acids

The main organic acids present in Kombucha include acetic, lactic, gluconic, as well as glucuronic acid [8]. The results of the study conducted by Kaewkod et al. (2019) showed that concentration of glucuronic acid (1.58 g/L), gluconic acid (70.11 g/L), D-saccharic acid-1,4-lactone (5.23 g/L), ascorbic acid (0.70 g/L), acetic acid (11.15 g/L) are higher in Kombucha obtained from black tea than in the case of oolong and green teas [105]. On the other hand, samples of green tea’s Kombucha were characterized by stronger antioxidant activities [105]. The health benefits of the presence of organic acids in Kombucha are antimicrobial activity, body detoxification, increased bioavailability of phenolic compounds, as well as affect on the hormonal balance.

Acetic acid (AA) is synthesized by AAB from ethyl alcohol by alcohol dehydrogenase and then aldehyde dehydrogenase. In the study conducted by Neffe-Skocińska et al. (2017) acetic acid concentration after 7 days of fermentation at 25 °C equaled 1.26 g/L [79], while Ivanišová and others noted 1.55 g/L after 7 days at 22 °C [71]. In addition to the different SCOBYs and incubation temperatures used in these two studies, attention should also be paid to the raw materials. In the case of first study 6 g/L (2 g/L of green tea and 4 g/L of black tea) and 100 g/L of sucrose was used, while Ivanišová and others used 30 g of white sugar and 5 g of black tea leaves. The sucrose content determines the activity of yeast and heterofermentative lactic acid bacteria, both groups of microorganisms responsible for the formation of ethyl alcohol during the fermentation of infusions. As a result of using lower sucrose concentrations for fermentation, low-alcohol or non-alcoholic products are obtained. However, it also affects acetic acid concentration. Finally, during long-term fermentation, after full utilization of saccharides and ethanol, acetic acid bacteria start to oxidize acetic acid, which results in a gradual decrease in the concentration of this organic acid [8].

Gluconic acid (GA) is naturally occurring compounds, which improves sensory properties of wine, vinegars, and honey. GA is the product of D-glucose oxidation by acetic acid bacteria, mainly *Gluconobacter oxydans*, as well as *Komagataeibacter xylinus* strains [106]. In general, it is considered that the fermentation starts from the oxidation of glucose to gluconic acid by *K. xylinus* [107]. In the 60-days study conducted by Chen and Liu (2000), the concentration of gluconic acid equaled 39 g/L. However, it was noted that production did not occur in the first days of fermentation [108].

Glucuronic acid (GlcUA) is produced by the oxidation of glucose by acetic acid bacteria belonging to the *Komagataeibacter* genus. Among the acids found in Kombucha, it is the acid with the strongest detoxifying properties [107]. The compound binds toxins present in the liver, which allows their effective excretion. What is more, glucuronic acid is a precursor of vitamin C. It takes part in glucuronization, increasing the availability of phenolic compounds, which in turn cause neutralization of free radicals, preventing the oxidation of polyunsaturated fatty acids. What is more, glucuronization also plays a significant role in hormone deficiencies and/or excesses of steroid hormones: increased solubility of steroids in water improves their bioavailability at low concentrations in the body, whereas in the case of high levels of steroid hormones, binding allows for excretion [8]. Significant differences in the concentrations of glucuronic acid in Kombucha are reported in the literature. Lončar et al. (2006) reported 0.016 g/L (21 days of fermentation), Jayabalan et al. (2007) obtained 1.71 g/L (18 days of fermentation), while Nguyen et al. (2015) obtained a concentration of 30.3 mg/L (5th day of fermentation, SCOBY without LAB) [57,98,109].

Lactic acid (LA) is a product of homo- or hetero LAB metabolism. The production of lactic acid from hexose and pentose by homofermentative strains (e.g., *Lactococcus* spp.) occurs via glycolysis pathway and pentose phosphate pathway, while heterofermentative strains (e.g., *Oenococcus* spp., *Leuconostoc* spp., *Levilactobacillus brevis*, *Limosilactobacillus fermentum*, *Limosilactobacillus reuteri*) use phosphogluconate pathways [110]. Lactic acid lowers the pH of the Kombucha, which contributes to the antimicrobial abilities. It has also has a positive effect on human health, improving blood circulation and preventing formation of blood clots [2].

D-saccharic acid-1,4-lactone (DSL) is considered to be the most healthful component in Kombucha [111]. The compound inhibits glucuronidase, an enzyme indirectly related with cancers; shows strong antioxidant properties and demonstrates an ability to reduce oxidative damage [112]; inhibits the apoptotic death of pancreatic β-cells [111]. DSL is a product of the glucuronic acid pathway, and it was found the presence of lactic acid bacteria positively influence the production of this compound by AAB belonging to *Gluconobacter* genus [113]. In general, the presence and concentration of the compound in final product is influenced by the type of infusion as well as SCOBY used to the fermentation [8].

### 3.4. Vitamins

The vitamins present in tea infusions are mainly low levels of B group vitamins, vitamins E, K and A, as well as vitamin C, which is present in green tea only [2]. Similar to phenolic compounds, vitamins exhibit strong antioxidant properties. Vitamin E prevents or delays the appearance of coronary artery disease and prevents cataracts. Vitamin C increase bioavailability of iron, prevents tooth decay, improves the body’s immunity. In turn, B vitamins counteract general fatigue, as well as prevent problems with concentration and memory [71]. During tea fermentation, the vitamin content increases significantly as a result of the metabolic activity of both acetic acid bacteria, lactic acid bacteria (if present) and yeasts. Vitamin C is recognized as the main vitamin in Kombucha. This strong antioxidant derives from glucose metabolism, particularly strains of the genus *Gluconobacter*. In general, synthesis of vitamin C from D-glucose involves six chemical steps and one fermentation (D-sorbitol oxidation) [104].

During Kombucha fermentation, B- complex vitamins can be synthesized by selected strains of lactic acid bacteria as wells as yeasts. Riboflavin (vitamin B*_2_*) is a precursor flavin mononucleotide (FMN) and flavin adenine dinucleotide (FAD), which acts as hydrogen carriers in biological redox reactions. The vitamin can be synthesized by selected strains of *Limosilactobacillus fermentum* (formerly *Lactobacillus fermentum*), *Lactiplantibacillus plantarum* (formerly *Lactobacillus plantarum*), *Lactococcus lactis* as well as *Leuconostoc mesenteroides* [114]. Thiamine (vitamin B_1_) is mainly involved in energy production, as well as show beneficial effect on the nervous system. *L. brevis* and *L. plantarum* are able to produce intracellular and extracellular vitamin B_1_ [115]. Folate (vitamin B_9_) is involved in cell metabolism (e.g., DNA replication, synthesis of nucleotides, amino acids). Lactic acid bacteria that produce different forms of folates include, among others selected strains of *Lactococcus lactis*, *Lactiplantibacillus plantarum*, and *Limosilactobacillus reuteri* fermentum (formerly *Lactobacillus reuteri*) [116]. In general, yeasts are sources of a B-complex vitamins such as B_1_, B_2_, B_5_, B_6_, B_7_, B_9_,B_12_, as well as ergosterol, which can be converted to vitamin D_2_. As in the case of lactic acid bacteria selected yeast strains belonging to *Z. bailii*, *S. cerevisiae*, can produce these bioactive compounds.

The concentrations of 0.74 mg/mL of vitamin B_1_, 0.08 mg/mL of vitamin B_2_, 0.52 mg/mL of vitamin B_6_, 0.84 mg/mL of vitamin B_12_ and 0.03 mg/mL were noted by Villarreal-Soto et al. (2018) [42]. On the other hand, 74 mg/100 mL of vitamin B_1_, 52 mg/100 mL of vitamin B_6_ and 84 mg/100 mL of vitamin B_12_ were found by Bauer-Petrovska and Petrushevska-Tozi (2000) in Kombucha obtained from black tea [117]. Malbaša et al. (2011) found that the concentration of vitamin C increase constantly, up to 28.98 mg/L on the 13th day of fermentation. In the same study authors found that “native SCOBY” more efficiently synthetized B*_2_* vitamin during fermentation of black and green teas than projected consortia of AAB + *Saccharomyces* sp. as well as AAB and *Zygosaccharomyces* sp. What is more, the synthesis of vitamin C was better during green tea fermentation, thus Kombucha based on green tea can be characterized by stronger antioxidant activity compared to the infusion of the green tea [78].

### 3.5. Bacteriocins

Due to the presence of Lactic Acid Bacteria bacteriocins, as well as bioactive peptides can be found in Kombucha. Bacteriocins, small protein structures, produced among others by LAB show an antagonistic and preventive effect against pathogenic and spoilage microorganisms. A highly valued property of bacteriocins is their safety, lack of cytotoxicity and their activity against specific group of microorganisms. Therefore, bacteriocins are relatively minimally invasive to the environment in which they operate. *Lactobacillus* spp. *Pediococcus* spp., *Lactococcus* spp, and *Leuconostoc* spp., strains are considered to be a main producers of bacteriocins among lactic acid bacteria. Bacteriocin that is most widely used in the food industry is nisin—a compound active against Gram-positive bacteria such as *Listeria monocytogenes*, *Staphylococcus aureus*, *Clostridium* spp., and *Micrococcus luteus* [118,119]. The activity of bacteriocins produced by *Leuconostoc mesenteroides* against *Staphylococcus aureus*, *Pseudomonas aeruginosa*, *Escherichia coli*, *Klebsiella pneumoniae* are known. Recently, bacteriocin, SLG10, was isolated from traditional eastern Chinese Kombucha [120].

## 4. The Effect of Storage

After the fermentation, filtration of the product and packaging in unit containers (bottles, cans), Kombucha may undergo further processes such as pasteurization to ensure microbiological stability of the product. Otherwise, it may lead to further changes in the composition by metabolic activity of SCOBY microorganisms. As a consequence, the antioxidant activity and health-promoting properties can be significantly reduced. Latest research results obtained by La Torre et al. (2021) show that after four months storage of black tea Kombucha tea at refrigerator, the phenolic content decreased significantly from the initial value of 234.1 ± 1.4 µgGAE/mL to 202.9 ± 2.1 µgGAE/mL, while pH increased from 2.82 to 3.16 [121]. However, it should be remembered that the effect of changes in the profiles of bioactive compounds and the decrease in antioxidant activity are the result of, among others, the activity of microorganisms. Hence, depending on the consortium, the changes in antioxidant properties may be different and should be investigated in detail.

## 5. Conclusions and Future Perspective

The health-promoting properties of the sweet-sour, sparkling Kombucha tea have been known for over 2000 years. As in the past, so now, tea leaves, sugar and the SCOBY consortium have been used in the fermentation of tea infusions. Due to the growing popularity of this drink, production processes and recipes are constantly being modified. It is caused by healthy food trends and consumer expectations towards products with increased sensory values, as well as products’ positive effect on human health.

Factors that essentially affect the antioxidant properties, and thus further health-promoting properties of these products, include the type of plant material, the conditions of tea brewing (time, temperature, type of water), the use of additives (juices, plant extracts), as well as composition of microorganisms in the SCOBY consortium. An important factor is also fermentation conditions (temperature, time, application of additional processes such as medium aeration). Despite the common belief that the consortium includes acetic acid bacteria, lactic acid bacteria and yeast, it is worth noting that the composition of SCOBY is not constant and is critically dependent on, e.g., the geographical region in which Kombucha is produced, as well as the factors of the fermentation. It is worth emphasizing that these processes should use microorganisms that have been previously characterized and identified, which is not a common practice.

As a result of metabolic activity, both AAB, LAB and yeast can synthesize health-promoting compounds, as well as take part in the hydrolysis of phenolic compounds derived from plant material. Despite the significant influence of microorganisms on the pro-health values of the product, a significant part of the products is obtained in processes in which the composition of the consortium has not been determined.

Moreover, in order to obtain products that will be characterized by strong health-promoting properties, it is necessary to extend the existing research on the influence of microorganisms’ activity on bioactive compounds, but also vice versa. The influence of bioactive compounds on the activity of microorganisms, also should be significantly expanded. In addition, the use of new strains that have not been present in Kombucha fermentation so far may have a positive effect on the product properties, both sensory, microbiological stability, and health-promoting activity. Despite the fact that this drink has been known for a long time, there is no doubt that works on improving this product should be continued.
